# A *TNXB* splice donor site variant as a cause of hypermobility type Ehlers–Danlos syndrome in patients with congenital adrenal hyperplasia

**DOI:** 10.1002/mgg3.1556

**Published:** 2020-12-17

**Authors:** Qizong Lao, Ashwini Mallappa, Fabio Rueda Faucz, Elizabeth Joyal, Padmasree Veeraraghavan, Wuyan Chen, Deborah P. Merke

**Affiliations:** ^1^ National Institutes of Health Clinical Center Bethesda MD USA; ^2^ The Eunice Kennedy Shriver National Institute of Child Health and Human Development National Institutes of Health Bethesda MD USA; ^3^ PreventionGenetics Marshfield WI USA

**Keywords:** CAH, congenital adrenal hyperplasia, EDS, Ehlers Danlos syndrome, *TNXB*

## Abstract

**Background:**

Congenital adrenal hyperplasia (CAH) due to 21‐hydroxylase deficiency is an autosomal recessive disease of steroidogenesis that affects 1 in 15,000. Approximately, 10% of the CAH population also suffer from CAH‐X, a connective tissue dysplasia consistent with hypermobility type Ehlers–Danlos syndrome (EDS). Most patients with CAH‐X carry a contiguous gene deletion involving *CYP21A2* encoding 21‐hydroxylase and *TNXB* encoding tenascin‐X (TNX), but some are of unknown etiology.

**Methods:**

We conducted clinical evaluation and medical history review of EDS‐related manifestations in subjects from two unrelated CAH families who carry a heterozygous *TNXB* c.12463+2T>C variant that alters the splice donor site of intron 42. A next generation sequencing (NGS) based EDS panel composed of 45 genes was performed for index patients from each family. TNX expression in patient skin biopsy tissues and dermal fibroblasts was assessed by qRT‐PCR and Sanger sequencing.

**Results:**

All three evaluated CAH patients carrying the *TNXB* splice site variant had moderate EDS manifestations. An NGS panel excluded involvement of other known EDS‐related variants. RNA assay on skin biopsies and dermal fibroblasts did not detect splicing errors in TNX mRNA; however, the removal of intron 42 was less efficient in the allele harboring the splice site variant as evidenced by the existence of a premature TNX RNA form, leading to an allele specific decrease in TNX mRNA.

**Conclusions:**

Carrying a *TNXB* c.12463+2T>C variant at the intron 42 splice donor site causes an allele specific decrease in TNX expression, which can be associated with moderate EDS in CAH patients.

## Graphical abstract

About 10% of congenital adrenal hyperplasia patients suffer from Ehlers‐Danlos Syndrome, a connective tissue dysplasia. In addition to previously reported contiguous CYP21A2‐TNXB deletions, here we report a TNXB splice site variant c.12463+2T>C as causative by decreasing the expression of tenascin‐X.

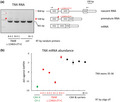

## 1 INTRODUCTION

Ehlers–Danlos Syndrome (EDS) includes a group of inherited connective tissue disorders with an estimated prevalence of 1:5000 and is typically caused by a spectrum of genetic defects mostly involved in collagen metabolism and pathways. EDS affects skin, joints, and vasculature with a broad range of symptomatology and is classified into six major subtypes (Beighton et al., 1998). The hypermobility type EDS (OMIM 130020) is the mildest but the most prevalent subtype, and its typical clinical manifestations include joint hypermobility, frequent joint dislocation, chronic joint pain, skin fragility, and less frequently, cardiac malformations. Unlike other EDS subtypes due to genetic defects affecting collagen pathways, the genetic etiology of hypermobility type EDS is largely unknown. However, a subset of subjects with hypermobility EDS have been identified to have defects in *TNXB*, which encodes tenascin‐X (TNX), a large glycoprotein essential for the constitution of the extracellular matrix (ECM) (Burch et al., 1997; Merke et al., 2013; Morissette et al., 2015; Schalkwijk et al., 2001; Zweers et al., 2003).

*TNXB* is mapped to the major histocompatibility complex on chromosome 6 (p21.33), with its last exon (exon 44) overlapping the 3’‐UTR of *CYP21A2*. Defects in *CYP21A2* lead to congenital adrenal hyperplasia (CAH) due to 21‐hydroxylase deficiency (CAH, OMIM 201910), an autosomal recessive disorder of steroidogenesis affecting cortisol biosynthesis with a prevalence of 1:15,000 for the classic or severe form and 1:200–1000 for the nonclassic or mild form (Hannah‐Shmouni et al., 2017; Speiser et al., 2018). The locus containing *TNXB* and *CYP21A2* is a low copy repeat region with several highly homologous genes, including nonfunctional pseudogenes. Approximately, 30% of CAH alleles are caused by unequal crossovers, termed 30‐kb deletions, resulting in chimeras with loss of *CYP21A2* functionality (Finkielstain et al., 2011). Some of the 30‐kb deletions generate chimeras of *TNXA*/*TNXB*, with complete removal of *CYP21A2* and replacement of the 3’ portion of *TNXB* with the corresponding portion of its highly homologous, but nonfunctional pseudogene *TNXA* (Burch et al., 1997). In fact, heterozygosity for a *TNXA*/*TNXB* chimera is the most common etiology of CAH‐X, the syndrome of EDS occurring in CAH patients (Chen et al., 2016; Merke et al., 2013; Morissette et al., 2015).

Approximately, 10% of CAH patients suffer from CAH‐X. To date, three *TNXA*/*TNXB* chimeras have been identified: CAH‐X CH‐1 causes haploinsufficiency due to a *TNXA* substitution for *TNXB* exons 35–44 (Burch et al., 1997; Merke et al., 2013); CAH‐X CH‐2 causes a dominant negative effect due to a *TNXA* substitution in *TNXB* exons 40–44 (Morissette et al., 2015); and CAH‐X CH‐3 has a *TNXA* substitution for *TNXB* exons 41–44 of unclear significance (Chen et al., 2016). It is also notable that although the two pathogenic CAH‐X chimeras (CH‐1 and CH‐2) cause EDS in an autosomal dominant manner regardless of the CAH status, the EDS manifestations are usually milder in CAH carriers compared to CAH patients, suggesting potential hormonal effects on the EDS phenotype (Merke et al., 2013; Morissette et al., 2015). Given the high prevalence of CAH‐X in the CAH population, we have expanded our clinical evaluation to cover EDS in an ongoing natural history study of CAH at the National Institutes of Health Clinical Center in Bethesda, Maryland (Clinical Trials # NCT00250159, cohort size >420 affected patients). Although the vast majority of CAH‐X cases are due to the pathogenic *TNXA*/*TNXB* chimeras, some patients with CAH are clinically affected by EDS of unknown etiology.

We hereby report a splice donor site variant in *TNXB* (NM_019105.8:c.12463+2T>C) that is associated with moderate EDS symptomatology in CAH patients. This variant changes the splice donor acceptor sites of intron 42 from a pair of canonical “GT‐AG” to noncanonical “GC‐AG,” leading to allele specific decrease in *TNXB* expression.

## 2 MATERIALS AND METHODS

This study was approved by the *Eunice Kennedy Shriver* National Institute of Child and Human Development Institutional Review Board. All adult subjects or parents of children (<18 years old) gave written informed consent. Clinical evaluations for diagnosis of CAH and EDS were completed as described previously (Merke et al., 2013). Genotyping of *CYP21A2* and *TNXB* exon 32–44, as well as the EDS screening by a next generation sequencing panel were completed by a CLIA‐accredited commercial laboratory (PreventionGenetics, LLC, Marshfield, WI) with previously described methodology (Chen et al., 2012, 2016). The haplotype of *TNXB* exons 35–44 was determined by TA cloning of a PCR product followed by standard Sanger sequencing (ABI Biosystems). TNX exons 35–44 was selected as the amplicon for RT‐PCR since exon 35 offers a 120 bp primer recognition window that is not present in the pseudogene *TNXA*, which is highly homologous to *TNXB*. Skin biopsies were obtained from selected subjects and were used to derive dermal fibroblast cells as described (Merke et al., 2013). Both the skin specimens and their derived dermal fibroblast cells were used for the RNA assays. NM_019105.8 and human genome assembly GRCh38/hg38 (NC_000006.12) were used as the TNX mRNA and *TNXB* DNA reference, respectively. Primers used for PCR assay are shown in Table 1. Details of the experimental materials and methods are available upon request.

**Table 1:** Primers used in this study

Amplicons	Primers (5′–3′)	Comments
*CYP21A2*‐*TNXB* exons 32–44	F: AGGTGGGCTGTTTTCCTTTCA R: CTGTGCCTGGCTATAGCAAGC	Amplifies *CYP21A2* and contiguous *TNXB* exons 32–44 for genotyping
*TNXB* and TNX exons 35–44	F: GTGACCGTGGTCTCGGTCCG	*TNXB* specific, no *TNXA* interference
	R: GTCATACTGGGGTGCGAGAGAGG	
*TNXB* and TNX exons 42–44	F: CAATGAGGCCCT GCACAGC	Also recognizes *TNXA* and its encoding noncoding RNA
	R: GTCATACTGGGGTGCGAGAGAGG	
TNX exons 35–36	F: GTGACCGTGGTCTCGGTCCG	For qRT‐PCR assay of TNX mRNA, no *TNXA* interference
	R:CACGCAACTGTGTGGGACCGTCAG	

## 3 RESULTS

**FIGURE 1:**
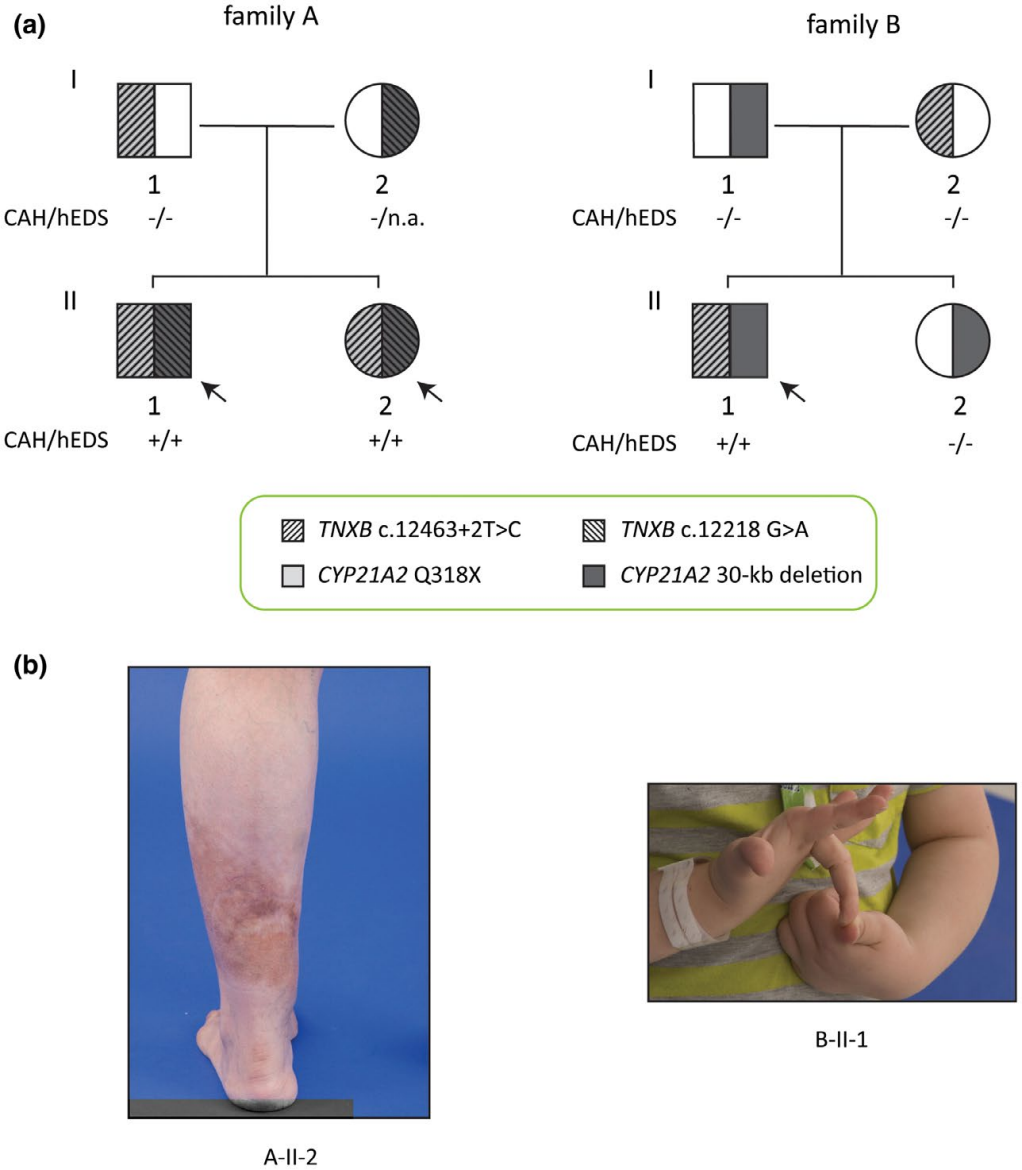
CAH‐X genetic pedigree and phenotype. (a) *TNXB* variants present in two unrelated families with CAH. Phenotypes of CAH and hypermobile EDS (hEDS) are presented in the pedigree. Plus (+) sign indicates present, negative (−) sign indicates absence, and n.a. indicates not available. Black arrows indicate subjects with phenotypic CAH‐X that are positive in both CAH and hypermobile EDS. (b) Examples of clinical EDS findings included thin skin and wide scars in subject A‐II‐2 and joint hypermobility in subject B‐II‐1

A total of five subjects from two unrelated families with CAH‐X of previously unknown etiology had a heterozygous *TNXB* c.12463+2T>C variant that is a “predicted loss‐of‐function” variant at splice donor site (Figure 1). In both families, this splice site variant shared an allele with a *CYP21A2* c.955C>T (p.Q318X) variant but not with a 30‐kb deletions variant. Except for a heterozygous *TNXB* c.12218G>A (p.Arg4073His) variant in family A, there were no other notable variants identified in these subjects by a next generation sequencing (NGS)‐based EDS panel composed of 45 genes, including all collagen related genes and *TNXB*. The absence of *TNXA*/*TNXB* chimeras (CAHX CH‐1 or CH‐2) was confirmed by PCR and Sanger methodology.

Four subjects carrying the *TNXB* splice donor site variant (three CAH patients and one CAH carrier) were available for clinical evaluation. All three CAH patients from the two families presented with a moderate EDS phenotype (Table 2, Figure 1). Two adult siblings (A‐II‐1 and 2) were found to have significant joint and skin manifestations; the pediatric patient (B‐II‐1) was noted to have joint hypermobility, spongy skin and minor cardiac defects including patent ductus arteriosus and patent foramen ovale; and all three had history of hernia. It is also notable that B‐II‐1 had a history of allergic disorders, which was suggested to be associated with *TNXB* by a genome wide association study (GWAS) (Portelli et al., 2015). The parents of family A were not available for an on‐site clinical evaluation but their medical records were available for review. A‐I‐1 had a history of bilateral inguinal hernias, degenerative joint disease, and allergy to kiwi fruit. A‐I‐2 had no EDS‐related medical history. The parents of family B were clinically evaluated. B‐I‐2 did not have generalized joint hypermobility (Beighton score 2) or abnormal skin findings. Her medical history was significant for back pain from degenerative spinal arthritis (diagnosed in her 20’s), chronic temporomandibular joint dysfunction and double‐jointed fingers with hypermobility of thumbs. B‐I‐1 did not have joint hypermobility or significant skin findings.

**Table 2:** Clinical characteristics of patients

	A‐II‐1	A‐II‐2	B‐II‐1
Sex/age (years)	M/44	F/46	M/3
Beighton score	0	2	6
Joint findings	Left shoulder dislocation x 2, chronic back pain, chronic left elbow and left hip pain; bilateral carpal tunnel syndrome	Chronic pain and stiffness in shoulders and knees, unilateral knee replacement	Generalized hypermobility
Skin findings	Thin skin, easy bruising, wide scars on back and leg, abdominal stria	Thin skin on shins with extensive hyperpigmented scars, easy bruising, multiple healed stria on thighs	Spongy skin
Cardiac findings	ECHO: mild tricuspid valve regurgitation, trace mitral and pulmonic valve regurgitation	ECHO: Focal thickening of the aortic valve leaflets with mild aortic regurgitation, mild tricuspid and pulmonic valve regurgitation, mildly elevated right atrial pressure	ECHO: patent ductus arteriosus, patent foramen ovale (noted at birth, resolved), trace mitral valve and tricuspid valve regurgitation
Additional clinical features	Mild chronic anemia, bilateral inguinal hernia, pes planus, recurrent plantar fasciitis, trigger finger, Meniere's disease, osteopenia, varicocele, gout, hemorrhoids	Mild chronic anemia, umbilical hernia, left anterior cruciate ligament repair, varicose veins, thyroid nodules, allergic to kiwi fruit (hives)	Umbilical hernia, chronic urticaria, seasonal allergies, allergic to cats and shellfish, lactose intolerance, speech delay, constipation

Abbreviations: ECHO, echocardiogram; F, female; M, male; MRI, magnetic resonance imaging.

**FIGURE 2:**
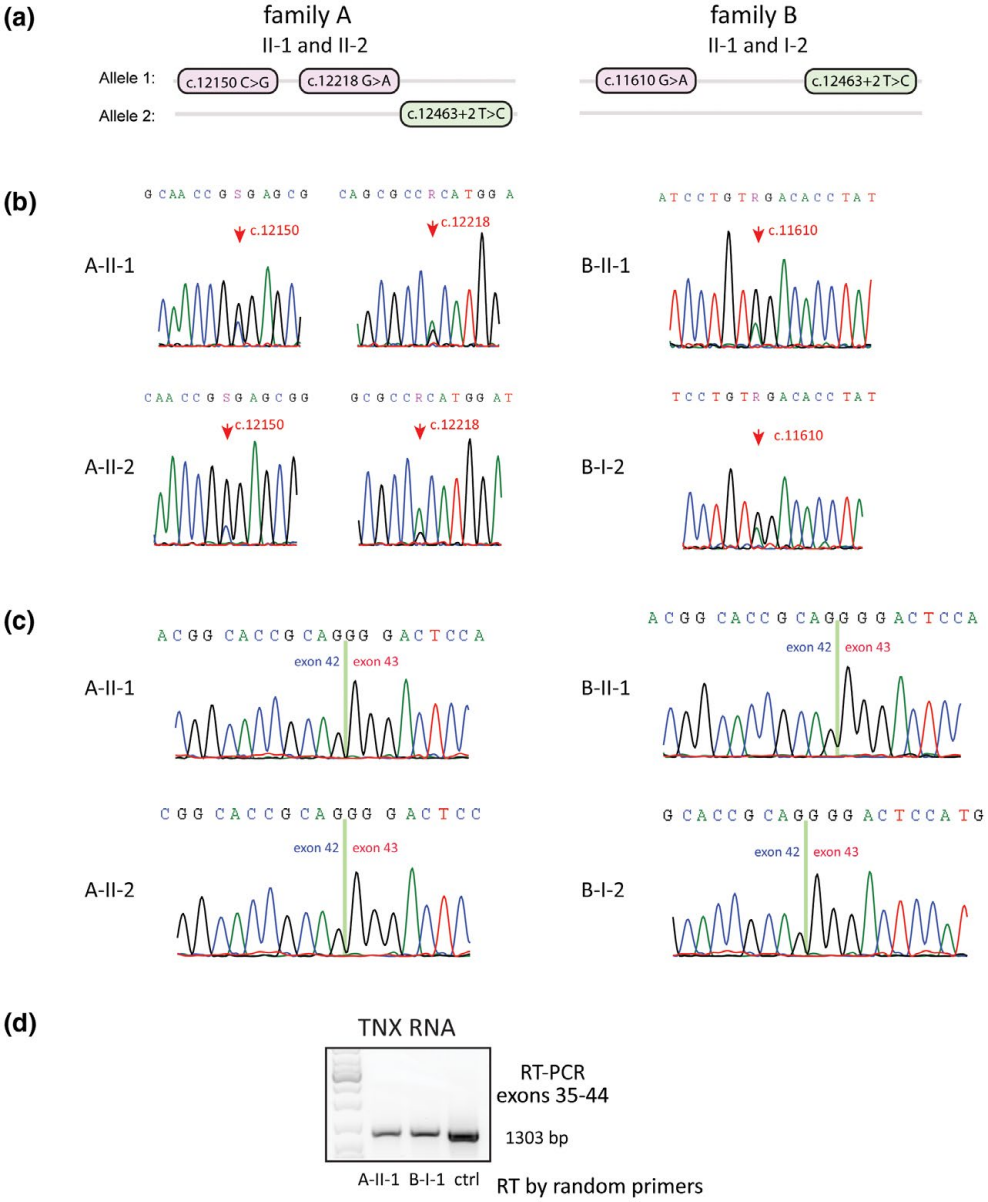
Effects of *TNXB* c.12463+2T>C (IVS42+2T>C) variant on tenascin‐X (TNX) expression in skin tissue. (a) Haplotypes of *TNXB* exons 35–44 locus of two carrier families are shown including the c.12463+2T>C variant (green oval) and other single nucleotide polymorphisms (SNPs; pink ovals). (b) Sanger chromatograms showing a decrease in TNX mRNA abundance from the allele of the splice site variant. RNA heterozygosity was confirmed in all subjects and red arrows mark the reduced chromatogram signals of the SNPs specific to the *TNXB* splice site variant alleles. (c) Sanger chromatograms of TNX mRNA exons 42–43 junction without a splicing error in all subjects. d RT‐PCR of TNX mRNA exons 35–44, clean single banded PCR products free of satellite bands suggested absence of alternative splicing or premature RNA. Subjects A‐II‐1 and B‐I‐1 are index for carrier and non‐carrier of *TNXB* c.12463+2T>C, respectively, and a commercial sourced adult skin total RNA sample is shown as a control. Skin RNA samples from all patients were extracted within 2 hours of skin biopsy

Five subjects (A‐II‐1, 2, B‐I‐1, 2, and B‐II‐1) underwent skin biopsy for the study of TNX expression. Their haplotypes of *TNXB* exons 35–44 locus are shown in Figure 2a as a reference for allele‐specific mRNA abundance study. RT‐PCR followed by Sanger revealed heterozygosity of TNX mRNA without detectable splicing error in all skin specimens bearing the *TNXB* c.12463+2T>C variant, however, mRNA from the variant alleles appeared less abundant than from the normal alleles as shown in Sanger chromatograms (Figure 2b,c). Examples of RT‐PCR product of TNX exons 35–44, which were the subjects of Sanger sequencing, are shown in Figure 2d. There were no detectable satellite bands that may represent splicing errors or alternative splicing.

**FIGURE 3:**
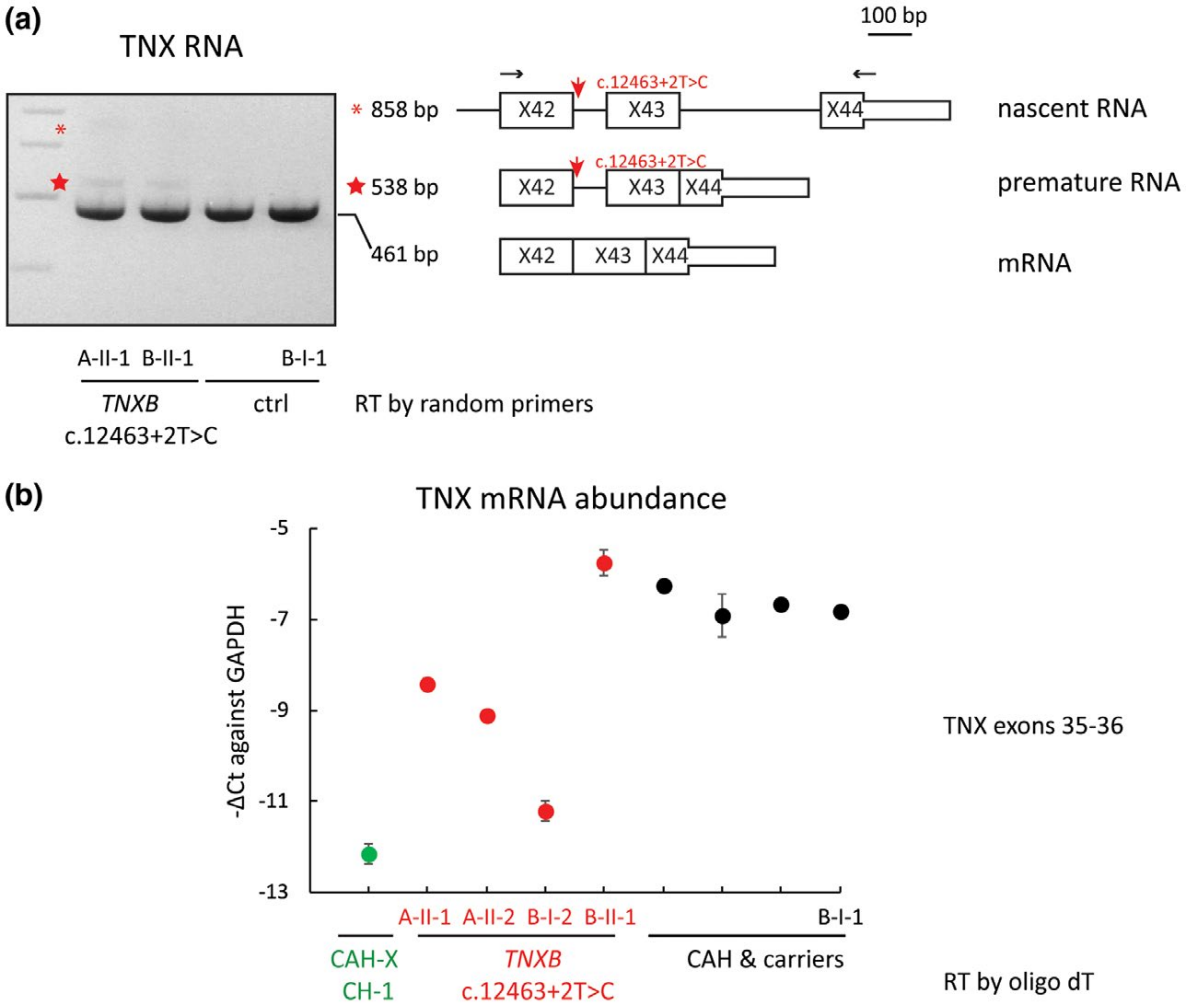
Effects of *TNXB* c.12463+2T>C (IVS42+2T>C) variant on tenascin‐X (TNX) expression in dermal fibroblasts. (a) RT‐PCR of TNX exons 42–44. Subjects A‐II‐1 and B‐II‐1 were selected as index for their respective families, Subject B‐I‐1 and an unrelated CAH patient were used as controls. A premature TNX RNA with moderate density (marked with red star) was found in the fibroblasts carrying the *TNXB* splice site variant but was absent from the control group; Sanger sequencing revealed that it failed to remove intron 42. A faint trace satellite band, revealed as nascent TNX RNA by Sanger (marked with red asterisk), was found in subject A‐II‐1. Both nascent and premature TNX forms were exclusively transcribed from the allele hosting the *TNXB* c.12463+2T>C variant (marked with red arrow), as revealed by Sanger. The primer recognition sites were marked with black arrows. RNA schemes are illustrated on the right. (b) A moderate decrease in TNX mRNA abundance commonly observed in dermal fibroblasts derived from *TNXB* c.12463+2T>C carriers. TNX mRNA abundance normalized to GAPDH are shown in three genotype categories: CAH‐X CH‐1 of TNX haploinsufficiency (green), CAH patient/carrier with *TNXB* c.12463+2T>C variant (red), CAH patients and carriers without *TNXB* defects (black). Normalization to other references, including β‐actin, α‐actin and α‐tubulin, obtained similar results (data not shown). Notably subject B‐II‐1 was 3 years old at the time of biopsy and there was no available age‐matched control

Those skin biopsies were then used to derive dermal fibroblasts. RT‐PCR of TNX exons 35–44 and Sanger analyses were repeated on total RNA samples extracted from early passage cells. Similar to the RNA findings in the skin biopsies, there was no detectable splicing error found in dermal fibroblasts; however, an allele–specific TNX mRNA decrease was observed (data not shown). Interestingly, using the fibroblast sourced RNA, RT‐PCR targeting TNX exons 42–44 detected a premature TNX form in the index samples of both families (Figure 3a). Sanger revealed that this premature RNA form failed to remove intron 42 (Figure 3a). A trace of nascent RNA was also detected in fibroblasts of subject A‐II‐1. Both premature and nascent RNA forms were transcribed exclusively from the allele bearing the *TNXB* c.12463+2T>C variant. Since the primers of RT‐PCR targeting TNX exons 42–44 also recognizes a homologous region of a noncoding RNA transcribed from *TNXA*, we sequenced the corresponding region of *TNXA* to eliminate it as the origin of the premature and nascent TNX RNA. This premature TNX RNA was found only in the fibroblasts and was not detectable in the skin tissues. We then assessed the overall TNX mRNA abundance in the fibroblasts of interest by qRT‐PCR with TNX exons 35–36 as the amplicon (no *TNXA* interference). A moderate decrease in TNX mRNA abundance was found in all dermal fibroblasts carrying the splice site variant except that of B‐II‐1, who was 3 years old at the time of skin biopsy. Three adult subjects (A‐II‐1, 2, and B‐I‐2) carrying the splice site variant had lower TNX mRNA abundance as compared to the non‐EDS CAH controls, but the loss of TNX mRNA was less severe than that of a subject carrying a heterozygous CAH‐X CH‐1 chimera of TNX haploinsufficiency (Figure 3b). Taken together, a heterozygous *TNXB* splice site variant c.12463+2T>C appeared to reduce the splicing efficiency at *TNXB* intron 42 by causing an allele‐specific decrease in TNX mRNA.

## 4 DISCUSSION

The *TNXB* c.12463+2T>C splice donor site variant reported here represents a novel and interesting cause of CAH‐X. Unlike the prior reports of CAH‐X which involve an extended 30‐kb deletion, the *TNXB* c.12463+2T>C variant alters the intron 42 splice donor‐acceptor site. Although there was no detectable splicing error in TNX mRNA, the heterozygous *TNXB* splice site variant c.12463+2T>C appeared to reduce the splicing efficiency at *TNXB* intron 42 leading to an overall moderate decrease in TNX mRNA abundance and a moderate EDS phenotype in the affected CAH patients. Normal *TNXB* intron 42 splice donor site “GT” is highly conserved among mammals. Although this splice site variant shared an allele with a *CYP21A2* p.Q318X variant in both studied families, due to the limited sample size, it remains unclear whether this *CYP21A2* genotype association is common or a random event. It is predicted as a high confidence “loss‐of‐function” variant with an allele frequency of 0.01096 by gnomAD browser (Lek et al., 2016).

RNA assays on skin biopsy specimens from the splice site variant carriers revealed no detectable splicing errors in TNX mRNA, but TNX mRNA was less abundant from the allele bearing the splice site variant. These findings were also seen in TNX mRNA from patient derived dermal fibroblasts. In those dermal fibroblasts, the premature TNX RNA that failed to remove intron 42 was found in the index samples of both families carrying the splice site variant, indicating a decrease in splicing efficiency at TNX intron 42. The premature TNX RNA form was found only in the affected fibroblasts but not in the skin tissues, likely due to more active TNX transcription in culture cells supplemented with fetal bovine serum that contains excessive growth factors and nutrition. Overall decrease in TNX mRNA abundance was observed in fibroblasts derived from all but one affected subject. The lone discordance observed in the fibroblasts of subject B‐II‐1 might be due to his young age or some differences in response to the cell culture supplements, for example, fetal bovine serum. Taken together, these results agree with the pathogenic prediction, and further suggest that the “loss of function” was in fact only partial and was likely due to a reduced splicing efficiency rather than erroneous splicing.

Subject B‐I‐2, who is a CAH carrier with the *TNXB* splice site variant, had only mild joint involvement despite having a low fibroblast TNX mRNA level. This is consistent with prior observations that CAH carriers with *TNXB* defects often have milder EDS manifestations compared to their affected CAH counterparts. The underlying steroidogenic defects due to CAH and/or hormone replacement therapy may play a crucial role in CAH‐X pathogenesis. Thus, a moderate decrease in TNX might be more likely to cause EDS in affected CAH patients than in CAH carriers or the general population. This might explain why this variant of interest has not been reported to be associated with EDS despite having an allele frequency of more than 0.01, since the prevalence of affected CAH is much lower (1:200 in nonclassic form or 1:15,000 in classic form) (Hannah‐Shmouni et al., 2017; Speiser et al., 2018). Other epigenetic factors might also influence TNX expression and phenotype.

Three single nucleotide polymorphisms (SNPs) were also observed in the *TNXB* exons 35–44 locus of the two families (two in family A and one in family B). Two of them (*TNXB* c.11610G>A and c.12150C>G) are synonymous. A *TNXB* c.12218G>A (p.Arg4073His) variant in family A is commonly predicted *in silico* as damaging or pathogenic by altering TNX protein folding (Chen et al., 2016), thus the CAH‐X phenotype in subjects A‐II‐1 and A‐II‐2 might be due to biallelic *TNXB* defects. However, it is also notable that the c.12218G>A variant mostly clusters with other pathogenic variants (c.12174C>G (p.Cys4058Trp), c.12514G>A (p.Asp4172Asn), and c.12524G>A (p.Ser4175Asn)) as part of the previously reported *TNXA*/*TNXB* chimeric genes, and family A subjects have been the only cases in our cohort to have an allele with this variant alone without clustering. Another issue is that this variant has an allele frequency of 0.02906 to 0.1221, which is higher than the reported population prevalence of EDS (Lek et al., 2016; National Center for Biotechnology Information, 2019). Therefore, it is unlikely that carrying the *TNXB* c.12218G>A variant alone is severe enough to cause phenotypic EDS. The mother in family A (A‐I‐2) was a heterozygous carrier of this variant; her medical history lacked EDS‐related conditions and she was not available for phenotyping.

The vast majority of human disease‐associated SNPs reside in the noncoding or intergenic regions of the genome, with about 45% in the intronic regions (Hindorff et al., 2009). While many intronic SNPs are associated with enhancers or promoters, those affecting pre‐mRNA splicing represent a primary link between genetic variants and diseases, accounting for an estimated one‐third of causative variants (Singh & Cooper, 2012). Although rigorous work has resulted in the development of many bioinformatic tools evaluating the mechanisms and regulation of pre‐mRNA splicing, it is still essential to evaluate splice site variants at the mRNA level in relevant tissues due to the complexity and tissue specificity of any given splicing event that demands the correct spliceosome to recognize the correct splice donor‐acceptor pair among many cryptic candidates. One interesting example is that a point mutation changing a “should‐be‐weak” donor of “AT” in the *SEDL* gene intron 4 to a “canonical” donor of “GT” leads to the erroneous splicing responsible for X‐linked spondyloepiphyseal dysplasia tarda (Xiong et al., 2009).

The *TNXB* splice donor site variant c.12463+2T>C changes the authentic splice donor acceptor from a canonical pair of “GT‐AG” to a noncanonical pair of “GC‐AG.” The former is more effective, common and accounts for >99% of splicing, while the latter is weak and accounts for 0.57–0.67% of splicing in mammals (Burset, Seledtsov, & Solovyev, 2000, 2001; Thanaraj & Clark, 2001). Both the consensus motifs of “GT” donor and “GC” donor are recognizable by U1 or U2 spliceosome for an accurate cleavage but the splicing process of “GC‐AG” pair is at lower speed (Aebi et al., 1986; Sibley et al., 2016). Variants of “T>C” at IVS+2 position changing splice donor site to non‐canonical “GC” have been reported in other genetic diseases, such as in *NP2* causing Niemann‐Pick C disease (Verot et al., 2007), and in *SCN5A* causing progressive cardiac conduction disorder (Schott et al., 1999). These variants mostly lead to alternative splicing or exon skipping. However, a unique aspect of the *TNXB* splice variant reported here that its lower splicing efficiency decreased mRNA abundance but did not cause a detectable splicing error. Residing at the second to last intron suggests little possibility for exon skipping or less of a chance for alternative splicing to occur. One limitation of our study is that the RNA assay was conducted only on skin related specimens and may not represent the splicing events occurring in other tissues. In addition, the premature TNX RNA which failed to remove intron 42 was detected only in culture dermal fibroblasts, but not in skin biopsy specimens.

In summary, carrying a *TNXB* c.12463+2T>C variant at the intron 42 splice donor site causes an allele specific decrease in TNX expression, which is associated with moderate EDS in CAH patients. The prevalence of CAH‐X among CAH patients is increasing as our understanding of *TNXB* variants expands. Further studies of *TNXB* and the influence of the hormonal milieu on TNX are needed, which will inevitably expand our understanding of connective tissue pathophysiology.

## CONFLICT OF INTEREST

D.P.M. received unrelated research funds from Diurnal Limited through the National Institutes of Health Cooperative Research and Development Agreement. The authors declare there is no conflict of interest in this work.

## AUTHOR CONTRIBUTIONS

Lab work was designed by Q.L. and was conducted by Q.L. and F.R.F.; Clinical work was planned and organized by A.M. and D.P.M. and clinical evaluations were done by A.M., E.J., P.V., and D.P.M. The manuscript was drafted by Q.L., A.M., and D.P.M with significant intellectual input by W.C. All authors approved the final version of the manuscript.
